# On the Transformation of Alkaline Sulphites in the Human System

**Published:** 1865-02

**Authors:** M. Carey Lea


### ON THE TRANSFORMATION OF ALKALINE SULPHITES IN THE HUMAN SYSTEM.

By M. CAREY LEA.

In connection with some interesting observations on the physiological effects of the alkaline sulphites when taken into the human system, as made by my friend Dr. W. F. Atlee, it has appeared to me that a brief account of a few chemical examinations made upon the same subject would not be altogether devoid of interest, especially as the indications are that these remedies are destined to play an important part in the relief of certain obscure and fatal forms of disease.

My first experiment was directed to observe, if possible, whether, after the administration of an alkaline sulphite, any free sulphurous acid was eliminated in the stomach; and if so, whether or not this would be reduced in that organ to the form of sulphydric acid or not.

As the gases set free in the stomach are carried off by the breath through the mouth, the following arrangement was adopted: a little very dilute ammonia was placed in a test-tube, and then, through a bent tube, the breath was driven through, in a period of from three to five minutes. The liquid was then tested by a lead-salt for HS, and afterwards with zinc and chlorydric acid for sulphurous acid, the hydrogen liberated being conducted over paper moistened with a lead-salt. Other portions were further examined by boiling with nitric acid and testing with chloride of barium, for sulphuric acid resulting from an oxidation of sulphurous acid.

The first trial was made two hours after swallowing thirtythree grains of bisulphite of soda. Neither the presence of $SO_2$, nor of HS could be detected by the foregoing methods.

In the second trial the sulphite was taken in a highly alkaline form. Thirty-three grains of monosulphite of soda in solution with twelve grains of bicarbonate of potash were swallowed, and three-quarters of an hour afterwards the breath was tested as before. No HS could be detected, but there were extremely faint traces of $SO_2$.

As respects the condition in which a sulphite would pass entirely through the human system, there could evidently be but two alternatives: it must either pass through unchanged, or must undergo oxidation and pass out as a sulphate.

In order to determine the point, the urine was examined. One hundred grains of monosulphite of soda were taken daily, in three doses of thirty-three grains each, and a large number of examinations were made of urine voided at various hours in the day. About two ounces were used in each trial, and the examination was made to ascertain whether any alkaline sulphite could be detected.

When the administration of the salt was first commenced, only very faint traces of sulphite could be detected, almost the whole appearing to be oxidized in the system; but on each succeeding day, the tests indicated an increasing quantity of sulphite, until large black stains were regularly obtained upon lead-paper, by exposure to the sulphydric acid obtained by the reduction of the sulphite.

In view of the powerful deoxodizing properties of the sulphites, it seemed probable that their administration would cause a depression of circulation, and observations were made to ascertain whether this would be the case. To afford a term of comparison, an observation was made on the state of the pulse two days before commencing the treatment:—

| | | Pulse at end of morning. | Pulse at end of afternoon. |
|---|---|---|---|
| Nov. 11. | Before the treatment was commenced...... | 78 | 73–4 |
| " 13. | Treatment commenced.......................... | 81 | 74 |
| " 14. | " continued.......................... | 81 | 75 |

The sulphite seems thus to have been without effect, or if any result was produced, it was rather a slight acceleration than a depression.

The conclusions to be drawn from the foregoing—at least so far as one case is capable of leading to conclusions—are that: 1st, when sulphite of soda is taken into the stomach, no sulphydric acid and a mere trace of sulphurous acid are evolved in the free state; 2d, that whilst the greater part of the sulphite is oxidized in passing through the system, some portion escapes this transformation, especially after the first few days of regular administration, and appears in the urine as unaltered sulphite; and 3d, it would seem to be indicated, *in this particular case*, that one hundred grains a day is as much, or more, than is capable of transformation in the system, and, therefore, of exercising its particular function, since even the whole of that was not found to have undergone oxidization. As the sulphite is often given with great advantage in much larger doses, I draw the last conclusion with great reservation, and not decisively. It seems probable (as indicated in a case which Dr. Atlee has mentioned to me, and which is published, p. 84) that very much larger doses are supported easily, and given with advantage. As the excess, if any, does not appear to be hurtful, I do not wish to be understood as arguing against the administration of the amount now deemed proper. This point, however, in its bearing upon actual practice, seems to deserve examination.

Further, the fact that a substance so readily oxidable as a sulphite, can be taken up by the circulation, and pass through it, unoxidized, as respects any portion of it, and be eliminated unchanged by the kidneys, appears to be very remarkable. It was, however, proved by too many separate and decisive experiments to be left in any doubt.

Finally, I may remark to those who may wish to repeat these experiments, that, unless proper precautions are taken, the reactions are easily missed, at least in those instances where the amount of unchanged sulphite voided is very small. My first trial was made on a few drachms of liquid, and I failed to get any reaction. On the following day, by using several ounces, I obtained a faint but unmistakable reaction, and this with each succeeding day became more and more pronounced. The materials should be placed in a flask with a narrow and very long neck. A small lump of zinc is to be introduced, and lastly a few drops of pure chlorhydic acid, enough only to cause a very slight action, accompanied by an almost imperceptible effervescence. A piece of bibulous paper, moistened with solution of acetate of lead, is to be inserted into the neck of the flask, so as almost to close it. The whole is then to be set aside for several hours, after the lapse of which the paper is to be examined for the characteristic stains of sulphide of lead.

It is to be observed that there are two forms in which sulphur may find itself in the urine, apart from any direct administration of substances containing it. It is always present in the form of saline sulphates. Upon these substances nascent hydrogen has no reducing effect. But sulphur is contained also in albumen, which in certain forms of disease may be present in the urine. The formula of albumen, according to Liebig, contains three equivalents of sulphur, amounting to nearly two per cent. It appeared to me to be a matter of interest to determine whether albumen was capable of giving up a portion of its sulphur to the reducing action of zinc and chlorhydric acid, and thus to settle the question whether its presence could embarass the investigation as to the presence of sulphites. A considerable quantity of albumen was introduced into a flask and exposed to the action of zinc and chlorhydric acid for many hours. An almost imperceptible stain was found upon the lead paper. Now the ingestion of one hundred grains of sulphite per day—less than the usual dose—I have found to be capable of charging the urine so heavily, with undecomposed sulphite, that lead paper is visibly darkened in a few seconds, and by longer exposure becomes perfectly black. Whilst, therefore, albumen seems not entirely incapable of acting upon lead paper, its influence is not such as to disturb the applicability of the test, inasmuch as an aqueous solution certainly containing ten times as much as could be found in a like bulk of albuminous urine, gave only almost imperceptible indications.—*Am. Jour. Med. Sciences.*

